# QHAWAY: An Instance Segmentation and Monocular Distance Estimation ADAS for Vulnerable Road Users in Informal Andean Urban Corridors

**DOI:** 10.3390/s26082569

**Published:** 2026-04-21

**Authors:** Abel De la Cruz-Moran, Hemerson Lizarbe-Alarcon, Wilmer Moncada, Victor Bellido-Aedo, Carlos Carrasco-Badajoz, Carolina Rayme-Chalco, Cristhian Aldana, Yesenia Saavedra, Edwin Saavedra, Alex Pereda

**Affiliations:** 1 Research Group GECOTRAN (Studies in Management, Construction and Transport), Faculty of Mining, Geological and Civil Engineering, Universidad Nacional de San Cristóbal de Huamanga, Ayacucho 05000, Peru; hemerson.lizarbe@unsch.edu.pe (H.L.-A.); victor.bellido@unsch.edu.pe (V.B.-A.); 2 Remote Sensing and Renewable Energy Laboratory (LABTELER), Faculty of Mining, Geological and Civil Engineering, Universidad Nacional de San Cristóbal de Huamanga, Ayacucho 05000, Peru; wilmer.moncada@unsch.edu.pe; 3 Biodiversity and Geographic Information Systems Laboratory (BioSIG), Faculty of Mining, Geological and Civil Engineering, Universidad Nacional de San Cristóbal de Huamanga, Ayacucho 05000, Peru; carlos.carrasco@unsch.edu.pe (C.C.-B.); carolina.rayme@unsch.edu.pe (C.R.-C.); 4 Research Group on Productive Efficiency, Environment and Society (EPAS UNF), Universidad Nacional de Frontera, Sullana 20100, Peru; caldana@unf.edu.pe (C.A.); ysaavedra@unf.edu.pe (Y.S.); 5 Universidad Nacional de Frontera, Sullana 20100, Peru; esaavedran@unf.edu.pe; 6 Statistics Programme, Faculty of Mining, Geological and Civil Engineering, Universidad Nacional de San Cristóbal de Huamanga, Ayacucho 05000, Peru; alex.pereda@unsch.edu.pe

**Keywords:** advanced driver assistance system, instance segmentation, monocular distance estimation, time-to-collision, YOLOv8, YOLO26, Andean urban environment, informal road infrastructure, mototaxi, road safety, vulnerable road users, Grad-CAM

## Abstract

Vulnerable road users in informal urban environments confront a distinct set of hazards that standard computer vision datasets are ill-equipped to represent: artisanal speed bumps constructed without regulatory compliance, deteriorated road markings, and the mototaxi—a three-wheeled motorized vehicle that constitutes the primary informal transport mode in intermediate Andean cities yet is absent from all major international repositories. This paper presents QHAWAY—from Quechua *qhaway,* a transitive verb meaning “to look; to observe”—an Advanced Driver Assistance System (ADAS) predicated on instance segmentation, monocular distance estimation via the pinhole camera model, and Time-to-Collision (TTC) computation, developed for the road environment of Ayacucho, Peru (2761 m a.s.l.), a city recognised by UNESCO as a Creative City of Crafts and Folk Art since 2019. A hybrid dataset comprising 25,602 images with 127,525 annotated instances across 12 classes was assembled by combining an original local collection of 4598 images (10,701 instances) captured through four complementary acquisition methods across the five urban districts of the Huamanga province with three established international datasets (BDD100K, BSTLD, RLMD; 21,004 images, 116,824 instances). A three-phase progressive training strategy with monotonically increasing resolution (640, 800, and 1024 pixels) was evaluated as an ablation study. A multi-architecture comparison spanning YOLOv8L-seg and the YOLO26 family (nano, small, large) identified YOLO26L-seg as the best-performing model, attaining mAP50 Box of 0.829 and mAP50 Mask of 0.788 at epoch 179. The integration of ByteTrack multi-object tracking with the pinhole equation $D=\left(H_{\mathrm{real}}\times f\right)/h_{\mathrm{px}}$ delineates operational risk zones aligned with the NHTSA forward collision warning standard (danger: <3 m; caution: 3–7 m; TTC threshold ≤ 2.4 s). The system sustains processing rates of 19.2–25.4 FPS on an NVIDIA RTX 5080 GPU. A systematic field survey established that 96% of the audited speed bumps fail to comply with MTC Directive No. 01-2011-MTC/14, constituting the first quantitative record of informal road infrastructure non-compliance in the Andean region. Validation was conducted under naturalistic driving conditions without staged scenarios. Grad-CAM explainability analysis, encompassing three complementary visualisation algorithms (Grad-CAM, Grad-CAM++, and EigenCAM), confirmed that model attention concentrates consistently on safety-critical objects.

## 1. Introduction

Pedestrians constitute the most acutely vulnerable group in road traffic: the World Health Organization reports that they account for 26% of global road fatalities, a proportion that surpasses 36% in low- and middle-income countries [1]. The Global Status Report on Road Safety 2023 recorded approximately 1.19 million annual deaths attributable to traffic crashes, representing an estimated economic burden equivalent to 3% of global GDP [1]. Critically, this toll is not uniformly distributed: nations with constrained road infrastructure budgets absorb 90% of fatalities despite accounting for only 60% of globally registered vehicles, and a disproportionate share of deaths involves informal or non-standard infrastructure that existing computer vision datasets are structurally unable to represent [1,2]. A systemic source of unrepresented road hazards in developing countries is informal road infrastructure: speed moderation devices constructed without adherence to official dimensional standards, unmarked pedestrian crossings, deteriorated road markings, and traffic actors absent from any major repository. In Peru, Directive No. 01-2011-MTC/14 issued by the Ministry of Transport and Communications (MTC) stipulates that speed bumps must not exceed 0.08 m in height, must present a minimum chord length of 3.50 m, and must be accompanied by vertical signage within a 100 m radius. The systematic field surveys conducted for the present study determined that 96% of the catalogued devices fail to satisfy at least one dimensional criterion of this regulation. This pervasive non-compliance generates invisible hazards both for drivers unfamiliar with a given route and for ADAS systems trained on standard road databases, wherein all moderation devices are regularised and properly signposted. The system presented in this paper is designated QHAWAY, a word drawn from Quechua—the principal indigenous language of the Andean region, spoken by an estimated 8–12 million people across six South American countries [3]. In the Southern Quechua dialect, of which the Ayacuchano variety is considered prototypical, *qhaway* is defined as a transitive verb meaning “to look; to observe” [4]. The derived causative form *qhawachiy* conveys the act of directing another’s gaze towards a hazard, a semantic dimension that aptly captures the anticipatory alerting function of the proposed system. The designation thus simultaneously evokes the local linguistic heritage of the study territory and the functional essence of the ADAS pipeline. In the Peruvian context, the National Road Safety Observatory (ONSV) reported 87,083 traffic crashes during 2023, resulting in 3316 fatalities [5]. Human factors account for 70.2% of incidents, with driver recklessness (28.2%) and excessive speed (26.6%) as the predominant causes. The department of Ayacucho recorded 549 traffic crashes in 2024, representing a year-on-year increase of 32.3%; 49.1% of departmental fatal crashes between 2021 and 2023 were concentrated in the province of Huamanga, with 62.1% occurring on roads devoid of horizontal markings [6,7]. The city operates at 2761 m above sea level in a high-ultraviolet-radiation environment with a road network characterised by colonial streets 4–6 m in width, topographic gradients reaching 12%, and a heterogeneous vehicle fleet dominated by the mototaxi—a three-wheeled motorised passenger transport vehicle that constitutes the primary informal transit mode in intermediate Andean cities—alongside conventional motorcycles, buses, and private vehicles [8]. Advanced Driver Assistance Systems (ADAS) incorporating deep-learning object detection have demonstrated tangible effectiveness in mitigating road hazards [9,10]. Instance segmentation extends bounding-box detection by recovering the precise pixel-level contour of each object, enabling spatial analyses unattainable with rectangular approximations: object boundary characterisation, zone-based proximity risk assessment, and pixel-wise overlap computation between traffic actors and infrastructure elements [11]. These capabilities are particularly consequential in informal road environments where the geometric profile of a moderation device (rounded versus trapezoidal cross-section) conveys safety-critical information beyond mere spatial location. Monocular distance estimation via the pinhole camera model offers a practical, sensor-agnostic alternative for resource-constrained ADAS deployment in developing countries [12,13]. The model requires only a calibrated focal length and the known object height to estimate longitudinal distance with a mean absolute error below 2.5 m over 1–30 m ranges, rendering it compatible with the TTC thresholds (≤2.4 s) established by NHTSA for forward collision warning systems [14,15]. Integration with multi-object tracking via ByteTrack [16] appends temporal velocity estimation for TTC computation, augmenting alert reliability beyond instantaneous distance thresholds. The localisation gap between international datasets and real deployment environments has been systematically quantified: Linardi et al. demonstrated that a model trained on the German GTSRB achieves 97.07% accuracy on its validation partition yet only 28.68% on genuine Indonesian images [17]. For Ayacucho, this gap is compounded by two additional factors absent from any major repository: the mototaxi as an independent vehicle class [18,19] and artisanal speed bumps whose dimensions systematically deviate from the standardised profiles present in BDD100K and nuScenes [20]. A survey of the pertinent literature reveals five research gaps motivating the present work. No computer vision dataset exists for the high-altitude Andean road environment with informal infrastructure. The phenomenon of non-standard artisanal speed bumps has not been quantified through metric surveys in any published ADAS study. Instance segmentation for zone-based pedestrian proximity risk assessment has not been applied in Latin American ADAS systems. The Peruvian mototaxi is absent from all international repositories, and no indexed publication reports instance segmentation metrics for this class. Finally, the concurrent presence of informal infrastructure and heterogeneous local vehicle fleets in a UNESCO-designated heritage urban context has not been addressed in the international computer vision or road safety literature [21,22,23]. This paper advances seven principal contributions. First, we construct a hybrid dataset of 25,602 images with 127,525 instances across 12 classes, incorporating original captures from Ayacucho obtained through four complementary acquisition methods and combined with three international datasets, with the mototaxi as a locally exclusive vehicle class. Second, we present a field survey documenting the dimensional non-compliance of informal speed bumps against MTC Directive No. 01-2011-MTC/14, providing the first quantitative record of this phenomenon. Third, we present an ADAS processing pipeline based on instance segmentation (YOLOv8L-seg + ByteTrack + pinhole model) operating at 1024 pixels. Fourth, we propose a three-phase progressive training strategy with increasing resolution (640, 800, 1024 px) evaluated as an ablation study. Fifth, we conduct a multi-architecture comparison across YOLOv8L-seg and the YOLO26 family (nano, small, large) [24], furnishing scaling insights for resource-aware ADAS deployment. Sixth, we present the first instance segmentation evaluation (mAP50 Box and mAP50 Mask) of the Peruvian mototaxi as an independent class in an indexed ADAS publication. Seventh, we conduct a Grad-CAM explainability analysis—encompassing three complementary visualisation algorithms—validating that model attention concentrates on safety-critical road objects.

## 2. Related Work

### 2.1. Instance Segmentation in ADAS Applications

Instance segmentation provides pixel-level delineation of individual objects, extending bounding-box detection with shape information critical for spatial analysis in driving scenarios. Pan et al. demonstrated that segmentation masks improve pedestrian proximity estimation compared with box-based methods in dense urban scenes [25]. Unar et al. applied visual attention mechanisms to instance segmentation for crosswalk occupancy analysis [11]. The segmentation variant of YOLOv8 has been specifically evaluated for real-time performance on embedded ADAS hardware [9].

### 2.2. Monocular Distance Estimation for ADAS

Monocular distance estimation without specialised sensors represents an active research frontier for cost-effective ADAS deployment. Dai et al. validated the pinhole model for pedestrian distance estimation, reporting mean absolute errors below 2.5 m for distances up to 30 m in urban scenarios [12]. Chaman et al. benchmarked YOLO-based monocular ADAS systems specifically for low-resource deployment contexts, confirming the applicability of the pinhole model with standard smartphone cameras [13]. Park et al. established the theoretical framework relating camera height, focal length, and apparent object height for road applications, which the present work extends to vehicle classes specific to the Andean urban context, including the mototaxi [15].

### 2.3. Speed Bump Detection

Speed bump detection has attracted increasing attention as a road safety and driver comfort application. Wang et al. proposed FPNet for visual perception of speed bumps in connected vehicles, attaining a detection accuracy of 91.3% on standardised datasets [20]. Peralta-López et al. combined a ZED stereo camera with deep neural networks for simultaneous speed bump and pothole detection [26]. Zhumadillayeva et al. integrated YOLO-CNN-LSTM for real-time road infrastructure monitoring [27]. Critically, all reviewed studies target standardised bumps conforming to regulatory dimensions; the informal artisanal devices prevalent in developing-country urban environments remain entirely unstudied.

### 2.4. Pedestrian Detection and Crosswalk Analysis

Pedestrian detection constitutes the highest-priority task for ADAS safety systems. Han et al. proposed a pedestrian detection framework integrating IoT and multi-sensor fusion for urban roads [28]. Kaya et al. applied Faster R-CNN and YOLOv7 to automatic crosswalk detection [21]. Zhang et al. developed CDNet for real-time crosswalk detection [29]. Wang et al. proposed CGADNet for simultaneous crosswalk and guide arrow detection [30]. Russon et al. evaluated pedestrian crossing safety using CNN models trained on aerial imagery [22].

### 2.5. Local Vehicle Classes and Data Localisation

The performance degradation of models trained on international datasets when applied to localised contexts has been rigorously documented. Linardi et al. demonstrated a 68 percentage-point drop when applying GTSRB-trained models to genuine Indonesian data [17]. Du et al. addressed local vehicle diversity with ML-E-YOLO for heterogeneous fleet detection [18]. Bao et al. proposed EMD-YOLOv8 for multi-class detection encompassing informal transport categories [19]. To the best of the authors’ knowledge, no study published in an indexed journal has reported instance segmentation metrics for the Peruvian mototaxi as an independent vehicle class.

## 3. Materials and Methods

### 3.1. QHAWAY ADAS System Architecture

**Figure 1:**
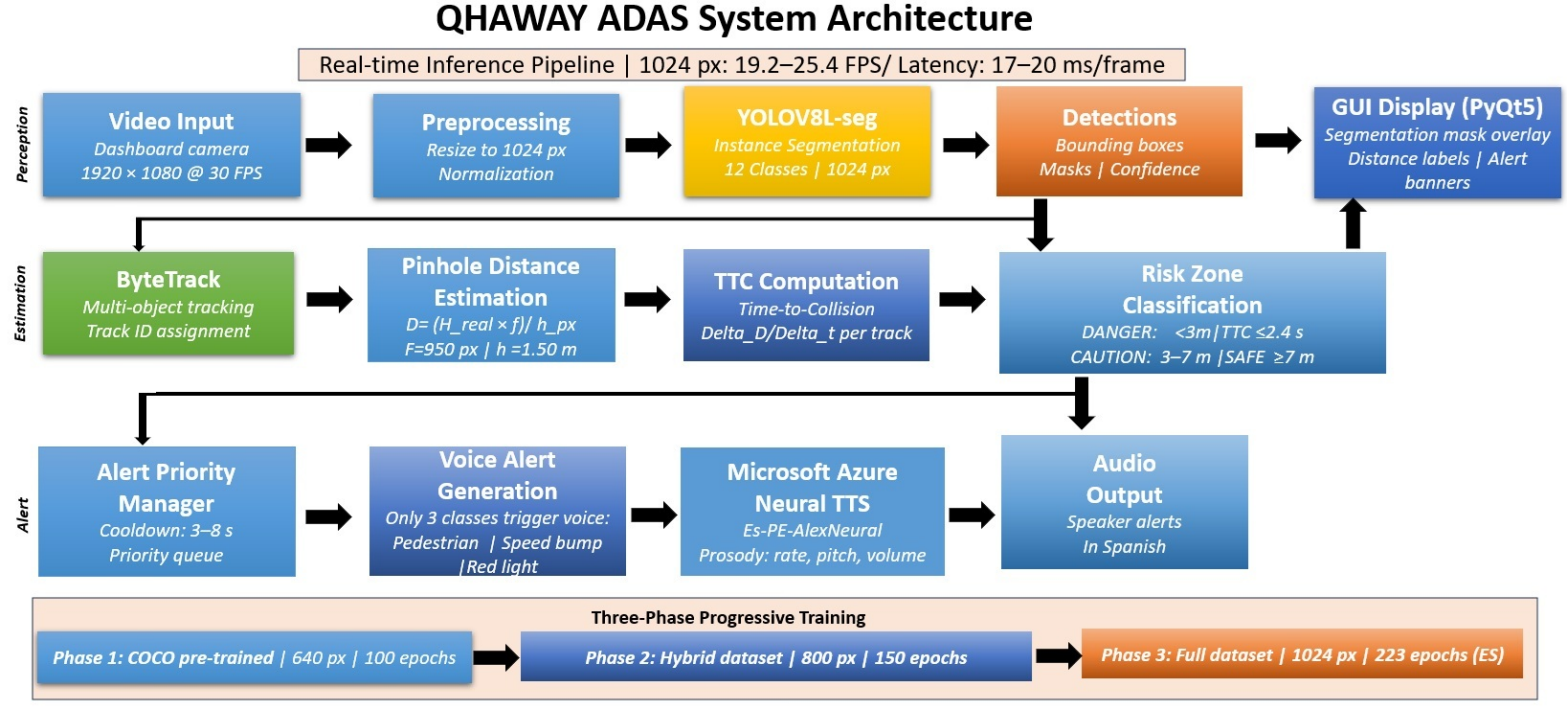
QHAWAY ADAS system architecture. The pipeline comprises five sequential modules: dashboard camera video input (1920×1080 px, 30 FPS) → YOLOv8L-seg instance segmentation at 1024 px → ByteTrack multi-object tracking → pinhole distance estimation and TTC computation → risk zone classification → priority-based voice alerts via Microsoft Azure Neural TTS (es-PE-AlexNeural). The graphical user interface (PyQt5) overlays segmentation masks, distance labels, and alert banners on the real-time video stream. The lower panel illustrates the three-phase progressive training strategy with increasing input resolution (Phase 1: 640 px, 100 epochs; Phase 2: 800 px, 150 epochs; Phase 3: 1024 px, 223 epochs; ES: Early Stopping).

The QHAWAY ADAS system integrates five functional modules into a unified real-time processing pipeline, as depicted in Figure 1.

*Capture module.* Frame acquisition is performed by a windshield-mounted smartphone camera operating at 1920×1080 pixel resolution and 30 frames per second.

*Detection and segmentation module.* YOLOv8L-seg processes each incoming frame and simultaneously generates bounding boxes, instance segmentation masks, and class confidence scores for the 12 defined categories. A detection confidence threshold of 0.50 was applied to suppress false positives—a deliberate design decision appropriate for an ADAS context in which spurious alerts erode driver trust and attentional compliance.

*Tracking module.* ByteTrack [16] assigns persistent temporal identities to each detected object, maintaining trajectory consistency across consecutive frames even under partial occlusion. The operative configuration was as follows: high-confidence threshold 0.50, low-confidence threshold 0.10, trajectory buffer of 60 frames, and association threshold 0.70.

*Distance estimation module.* The pinhole camera model [12,15] was implemented to estimate the longitudinal distance between the vehicle and each detected object as follows:

(1) $$D=\frac{H_{\mathrm{real}}\times f}{h_{\mathrm{px}}}$$

where *D* denotes the estimated distance in metres, $H_{\mathrm{real}}$ is the known real height of the object class, *f* is the camera focal length in pixels, and $h_{\mathrm{px}}$ is the apparent object height in the image in pixels. Parameters were calibrated for the Toyota Hilux (Toyota Motor Corporation, Toyota, Japan) mounting configuration: camera height $h_{\mathrm{cam}}=1.50$ m, focal length f=950 px, and optical axis tilt angle θ=5°. Reference real heights were established per class from systematic field measurements (Table 1).

For person detection (class 4), a complementary ground-contact-point estimation was computed via perspective geometry:

(2) $$D_{\mathrm{foot}}=\frac{h_{\mathrm{cam}}}{\tan\;\left(\;\arctan\;\left(\frac{y_{\mathrm{bottom}}-c_{y}}{f}\right)+\theta \right)}$$

where $y_{\mathrm{bottom}}$ is the pixel row of the bounding box lower edge and $c_{y}$ is the optical centre row. The final distance estimate for persons was computed as the mean of Equations (1) and (2), providing dual-source robustness against height-estimation errors.

The Time-to-Collision for trajectory *i* at time *t* is estimated as:

(3) $${\mathrm{TTC}}_{i}\left(t\right)=\frac{D_{i}\left(t\right)}{v_{i}^{\mathrm{approach}}}$$

where $v_{i}^{\mathrm{approach}}$ denotes the mean approach velocity derived from the distance history of trajectory *i*. Objects with $v_{i}^{\mathrm{approach}}\leq 0.5$ m/s do not activate TTC-based alerts.

*Alert module.* Drawing upon the NHTSA forward collision warning threshold [14], two operational zones were defined: a danger zone (D<3.0 m or TTC < 2.5 s, immediate voice alert) and a caution zone (3.0≤D<7.0 m, preventive alert). Objects at D≥7.0 m are rendered without voice output. Zone membership is evaluated exclusively for objects detected within the central lane region (20%–80% of frame width), minimising false alerts from stationary roadside elements. Three classes activate voice synthesis: person (distance- and TTC-conditioned), speed bump (contact-point-based, within 30 m), and red traffic light (unconditional). Voice output was implemented via Microsoft Azure Neural TTS (es-PE-AlexNeural) [32] with a priority-based cooldown scheduler.

### 3.2. Study Area

**Figure 2:**
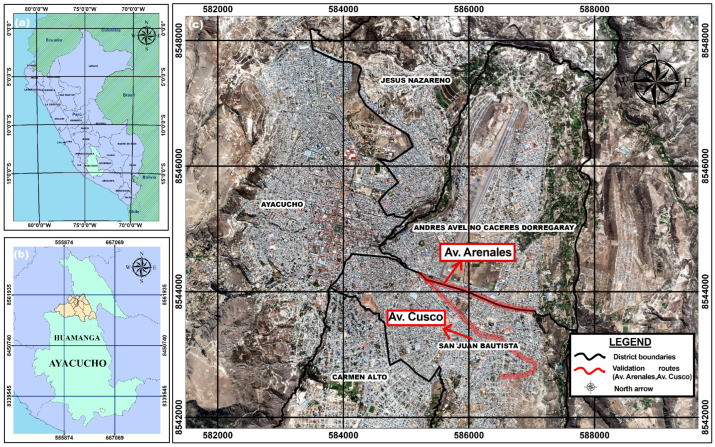
Study area. (**a**) Location of Peru with the Ayacucho department highlighted. (**b**) Huamanga province showing the five urban districts where data collection was conducted. (**c**) Urban area with the operational validation routes (Av. Arenales and Av. Cusco) highlighted. Coordinate values in panels (**b**,**c**) correspond to UTM projected coordinates (WGS84, Zone 18S) expressed in metres; no thousands separator is applied following cartographic convention.

The study was conducted across the urban territory of Huamanga province, department of Ayacucho, Peru, situated at 2761 m above sea level on the eastern flank of the Andes. The study area encompasses five contiguous districts forming the urban conurbation: Ayacucho, San Juan Bautista, Carmen Alto, Jesús Nazareno, and Andrés Avelino Cáceres Dorregaray. The Ayacucho district hosts the Historic Centre, declared a National Cultural Heritage Site by the Ministry of Culture of Peru and recognised by UNESCO as a Creative City of Crafts and Folk Art since 2019. This centre is characterised by narrow colonial streets with carriageway cross-sections of 4–6 m, adobe and stone architecture, and intense pedestrian and commercial activity. The peripheral districts exhibit a more dispersed urban fabric with wider arterial avenues, where public transport and freight traffic predominates. Data collection traversed multiple roads across all five districts, including Avenidas Arenales, Cusco, 9 de Diciembre, Independencia, Mariscal Cáceres, Las Américas, and República de Venezuela, as well as various streets in the historic centre, the environs of the Universidad Nacional de San Cristóbal de Huamanga (UNSCH), and the Plaza de Armas. Operational validation of the QHAWAY ADAS system was conducted specifically along Av. Arenales and Av. Cusco, which traverse the Ayacucho and San Juan Bautista districts and constitute high-traffic arterial segments. The road safety context is critical: 49.1% of departmental fatal crashes between 2021 and 2023 [6] were concentrated in Huamanga province, with 62.1% occurring on roads devoid of horizontal markings [7]. Figure 2 presents the geographic location of the study area.

### 3.3. Informal Speed Bump Field Survey

Prior to dataset construction, a systematic metric field survey of speed moderation devices was conducted along the study avenues and across the broader urban road network. For each catalogued device, the following data were recorded: GPS coordinates via the GPS Map Camera application (which stamps geographic coordinates, date, and time on each photograph), height, chord length, paint condition, presence or absence of vertical signage within a 60 m radius, and georeferenced photographic documentation. Evaluation criteria were drawn from MTC Directive No. 01-2011-MTC/14, which stipulates a maximum height of 0.08 m, a minimum chord length of 3.50 m, trapezoidal cross-sectional geometry, and mandatory prior vertical signage within 100 m.

**Figure 3:**
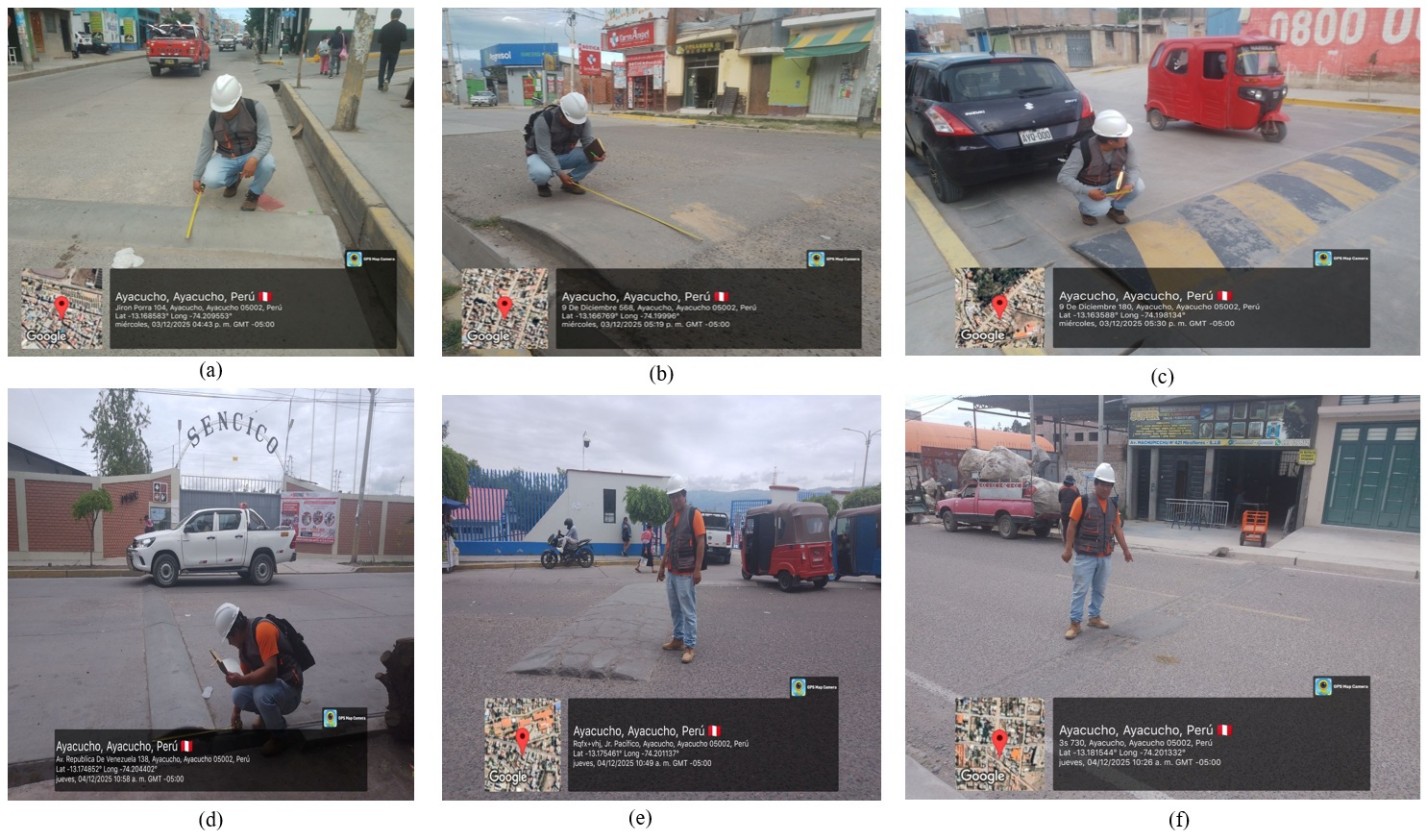
Field survey of informal speed bumps with georeferenced photographs captured via GPS Map Camera: (**a**) height measurement at Jr. Porra 104, Ayacucho district, revealing a non-regulatory semicircular profile; (**b**) dimensional measurement at 9 de Diciembre 588, with height exceeding the 0.08 m regulatory limit; (**c**) speed bump with partially deteriorated zebra-stripe paint at 9 de Diciembre 180, with a mototaxi circulating in proximity; (**d**) measurement at Av. República de Venezuela 138 (SENCICO area), Jesús Nazareno district; (**e**) speed bump partially destroyed by local residents, evidencing the conflict between the perceived need for speed control and deficient technical implementation; (**f**) researcher documenting a degraded speed bump lacking prior vertical signage. GPS Map Camera watermarks display georeferenced coordinates (latitude/longitude), acquisition date, and local time (GMT−05:00). Negative coordinate values correspond to the Southern and Western Hemisphere conventions; these watermarks are automatically embedded by the application and cannot be modified. Street names correspond to official Peruvian toponyms.

Table 2 summarises the survey findings, and Figure 3 presents photographic documentation of the catalogued devices. Of the total surveyed population, 96% deviated from at least one dimensional criterion; the predominant non-conformances were semicircular cross-sections (as opposed to the regulatory trapezoidal profile), heights exceeded the 0.08 m limit, and there was an absence of associated vertical signage. Cases of speed bumps partially destroyed by residents were also documented, evidencing the inherent conflict between the perceived need for speed control and the deficient technical implementation of these devices.

### 3.4. Hybrid Dataset Construction

#### 3.4.1. Data Sources and Collection Methods

**Figure 4:**
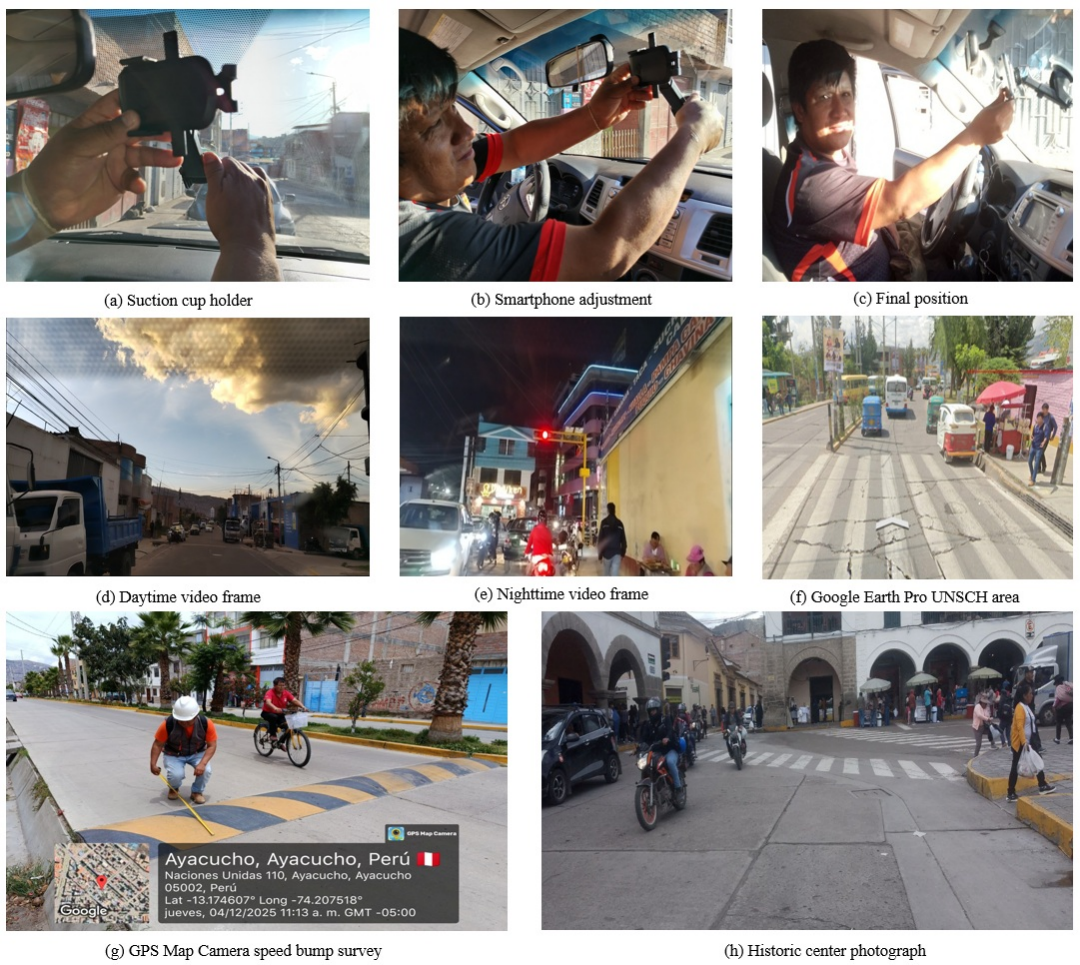
Data collection methods for the local Ayacucho dataset: (**a**) suction-cup holder mounted on the Toyota Hilux windshield; (**b**) adjustment of the Redmi Note 12 Pro smartphone on the holder; (**c**) final device position at 1.50 m height for Full HD video capture (1920 × 1080 px, 30 fps); (**d**) daytime video frame extracted from the moving vehicle; (**e**) nighttime video frame with traffic lights and pedestrian activity; (**f**) Google Earth Pro (version 7.3.7.1155) capture in Street View mode in the area surrounding UNSCH; (**g**) georeferenced photograph with GPS Map Camera during the speed bump field survey, displaying stamped coordinates, date, and time; (**h**) conventional photograph of a historic centre intersection with mixed traffic (motorcycles, mototaxis, pedestrians).

Dataset construction followed a hybrid strategy combining local field collection and publicly available international sources. Figure 4 illustrates the four acquisition methods employed.

*Local sources (4598 images, 10,701 instances).* Local images were acquired through four complementary methods across the five urban districts of Huamanga province. The first method comprised video recordings from a moving vehicle Toyota Hilux (Toyota Motor Corporation, Toyota, Japan) equipped with a Redmi Note 12 Pro smartphone (Xiaomi Corporation, Beijing, China) (108 MP native sensor; video recorded in Full HD mode at 1920×1080 pixels, 30 fps) mounted via a suction-cup holder on the windshield at 1.50 m height. Recordings were made along multiple routes under both daytime and nighttime conditions, and representative frames were extracted at regular intervals. The second method employed the GPS Map Camera application, which automatically stamps geographic coordinates, date, and time on each photograph, furnishing a georeferenced record particularly valuable for speed bump documentation. The third method involved conventional photographs captured in the historic centre, the UNSCH campus, and various urban intersections. The fourth method utilised Google Earth Pro (version 7.3.7.1155, Google LLC, Mountain View, CA, USA) images in Street View mode and street-level captures, enabling coverage of extensive urban areas including zones with restricted vehicular access.

*International sources (21,004 images, 116,824 instances).* Three publicly accessible datasets were incorporated: BDD100K [33] (heterogeneous urban driving, Berkeley, CA, USA), the Bosch Small Traffic Lights Dataset (BSTLD) [34], and the Road Lane Marking Dataset (RLMD) [35]. These sources contributed images for classes shared with the local collection (person, car, motorcycle, truck, bus, traffic light, road markings), broadening the variability of illumination conditions, viewpoints, and traffic scenarios to enhance model generalisation.

#### 3.4.2. Class Definition

Twelve representative classes of road elements critical to driver safety in the Ayacucho context were defined. Selection was governed by three criteria: frequency of occurrence in the local road environment, relevance to driver safety, and absence or systematic underrepresentation in international datasets. Table 3 presents the class distribution in the local subset.

#### 3.4.3. Final Hybrid Dataset

**Figure 5:**
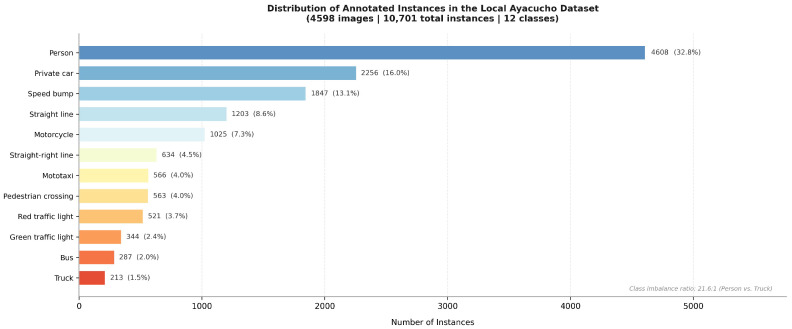
Instance distribution by class in the QHAWAY hybrid dataset, differentiating contributions from the local Ayacucho collection and the international datasets.

The hybrid dataset aggregates both source collections into a total of 25,602 images with 127,525 annotated instances across 12 classes. The corpus was partitioned as follows: 70% for training (17,921 images), 20% for validation (5120 images), and 10% for testing (2561 images). Table 4 summarises the composition, and Figure 5 presents the class distribution.

### 3.5. Annotation with LabelMe and SAM2

**Figure 6:**
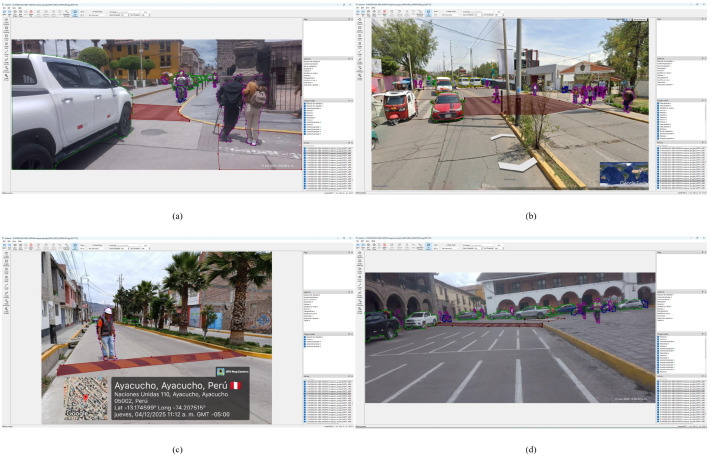
Representative annotation examples from the local Ayacucho dataset illustrating the four data acquisition methods employed: (**a**) conventional photograph captured on a narrow colonial street in the historic centre of Ayacucho, showing polygon annotations for a person with reduced mobility and an accompanying pedestrian on a deteriorated crosswalk; (**b**) Google Earth Pro (version 7.3.7.1155, Google LLC, Mountain View, CA, USA) street-level capture near UNSCH with SAM2-assisted segmentation of mototaxis—the locally dominant informal transport mode absent from international repositories; (**c**) georeferenced photograph acquired via GPS Map Camera (lat −13.1746°, long −74.2075°, 4 December 2025) documenting a non-conforming speed bump with trapezoidal annotation; (**d**) historic centre scene annotated from a conventional photograph, capturing high-density pedestrian and vehicular activity at the Plaza de Armas. All polygon masks were generated via SAM2 prompt-assisted segmentation and manually verified by the annotator.

Instance segmentation demands pixel-level polygon annotations. LabelMe [36] was selected as the annotation platform for its native polygon annotation support, seamless integration with SAM2 [37,38], and capacity for local offline execution. Annotation followed the SAM2-assisted workflow: the annotator placed a prompt point within each object of interest, SAM2 generated an initial polygon mask, and the annotator subsequently refined contour details prior to confirming the class label. Annotations were exported in YOLO format with normalised segmentation polygon coordinates. A rigorous quality control protocol included independent cross-review of 20% of annotations by a second evaluator, exclusion of low-quality frames (motion blur, severe occlusion exceeding 80%), and systematic label consistency verification. Figure 6 presents representative annotation examples from the local Ayacucho dataset illustrating the SAM2-assisted workflow across diverse road scenarios.

### 3.6. YOLOv8L-seg Architecture

YOLOv8L-seg [39] was selected as the primary detection model. The architecture integrates a CSPDarknet backbone with C2f modules, a PAN (Path Aggregation Network) neck for multi-scale feature fusion at three resolutions (80×80, 40×40, 20×20), anchor-free detection heads, and a dedicated segmentation head that produces per-instance binary masks. With 43.7 M parameters, YOLOv8L-seg affords substantially greater representational capacity than the medium variant (25.9 M) whilst sustaining real-time performance at 1024 pixels—a resolution deliberately selected to preserve fine contour details of small objects such as road markings and distant mototaxi silhouettes.

### 3.7. Three-Phase Progressive Training Strategy

Training was performed on an NVIDIA GeForce RTX 5080 GPU (16 GB VRAM) employing a three-phase progressive strategy with monotonically increasing input resolution, designed to maximise performance within a constrained computational budget. Table 5 summarises the configuration of each phase.

*Phase 1 (640 px):* The COCO-pretrained [40] YOLOv8L-seg model was fine-tuned at 640 pixels with a batch size of 16, enabling rapid initial convergence and adaptation of the detection and segmentation heads to the 12 target classes.

*Phase 2 (800 px):* Resolution was elevated to 800 pixels from the best Phase 1 checkpoint, with a reduced learning rate. This phase permitted the model to refine medium-scale object detection and adapt low- and mid-level feature representations to the specific visual properties of the Andean urban environment.

*Phase 3 (1024 px):* Resolution was further elevated to 1024 pixels with additional learning rate reduction and early stopping with a patience of 50 epochs. This phase yielded the most substantial improvement in small and distant object detection as well as mask contour precision; training executed 223 epochs with the best checkpoint selected at epoch 173.

Data augmentation techniques applied throughout training comprised horizontal flip (probability 0.5), mosaic composition (probability 1.0 in phases 1–2, disabled in phase 3), rotation (±10°), scale (0.5–1.5), and HSV colour jitter (H = 0.015, S = 0.7, V = 0.4). Mosaic augmentation was deactivated in phase 3 in accordance with the recommendation of Jocher et al. for high-resolution training regimes [39].

### 3.8. Grad-CAM Explainability Analysis

To validate the semantic coherence of the learned representations, a comprehensive explainability analysis was conducted on the final YOLOv8L-seg model employing three complementary visualisation algorithms from the Grad-CAM family: the original Grad-CAM [41], which computes spatially weighted channel activations from first-order gradients; Grad-CAM++ [42], which extends the weighting scheme using second- and third-order gradient moments to improve localisation under multiple class instances; and EigenCAM [43], which derives the attention map from the principal component of the feature tensor via singular value decomposition, without requiring gradient computation. Attention maps were extracted from the SPPF layer (layer 9) of the backbone, which captures the most semantically abstract feature representations. Representative scenes from the Ayacucho dataset were analysed, covering mixed-traffic scenarios with speed bumps, pedestrians, and diverse vehicles.

## 4. Results

### 4.1. Training Progress

**Figure 7:**
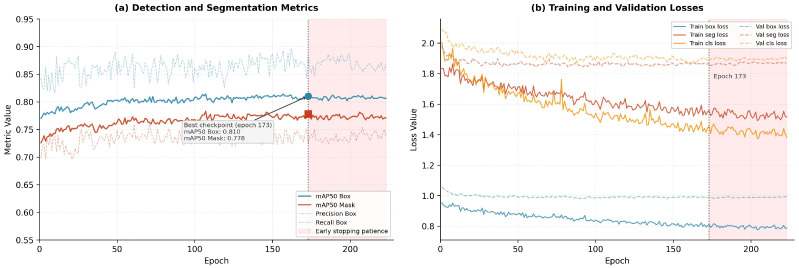
Phase 3 training curves (1024 px, 223 epochs). (**a**) Evolution of mAP50 Box and mAP50 Mask per epoch. (**b**) Training and validation loss curves. The optimal checkpoint was reached at epoch 173.

Figure 7 presents the training progression during Phase 3 (1024 px, 223 epochs). Detection and segmentation metrics stabilised near epoch 173, which was selected as the optimal checkpoint by the early stopping criterion. The convergent behaviour of training and validation loss curves provides no substantive evidence of overfitting.

### 4.2. Three-Phase Ablation Study

The three training phases were evaluated independently to quantify the marginal contribution of each resolution increment. Table 6 presents the results.

Phase 3 produced the largest mask improvement (+14.5 pp), corroborating that 1024-pixel resolution is especially beneficial for contour delineation of geometrically complex shapes (speed bump profiles, mototaxi silhouettes). The cumulative gain of +24.9 pp in mAP50 Mask validates the progressive training design as an effective strategy for instance segmentation under limited computational resources.

### 4.3. Overall Detection and Segmentation Performance

Table 7 presents the aggregate performance of YOLOv8L-seg at epoch 173.

The model attained mAP50 of 0.810 for detection and 0.778 for segmentation. The precision of 0.885 indicates a low false positive rate, while the recall of 0.724 reflects that approximately 72% of present objects were correctly identified—an acceptable trade-off for an ADAS application in which false alert suppression is a primary design objective.

### 4.4. Per-Class Performance

Table 8 details performance across the 12 classes. The highest-performing class was speed bump (mAP50 Box = 0.923, mAP50 Mask = 0.897), a particularly consequential result given its central practical importance. The mototaxi class attained mAP50 Box of 0.769 and mAP50 Mask of 0.738—a notable outcome considering that this category is absent from all international datasets and was trained exclusively on locally collected images. The lowest-performing class was straight line (mAP50 Box = 0.610), attributable to limited visual variability and the relatively small number of validation instances (109).

### 4.5. Precision–Recall Curves

**Figure 8:**
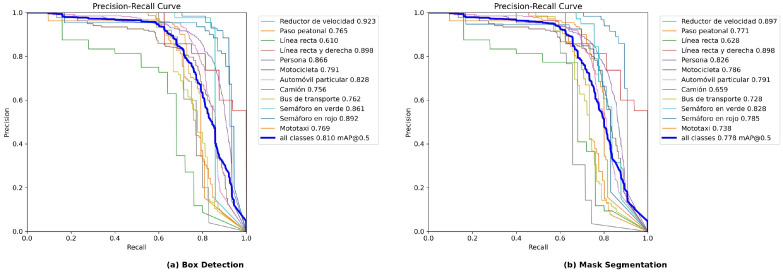
Precision–recall curves of YOLOv8L-seg at epoch 173. (**a**) Box detection (mAP50 = 0.810). (**b**) Mask segmentation (mAP50 = 0.778). Class labels retain the original Spanish-language nomenclature from the annotated dataset.

Precision–recall curves for both the detection and segmentation tasks are presented in Figure 8. The area under each curve corroborates the mAP50 values of 0.810 (Box) and 0.778 (Mask) reported in the aggregate metrics.

### 4.6. Multi-Architecture Comparison: YOLOv8L-seg vs. YOLO26 Family

**Figure 9:**
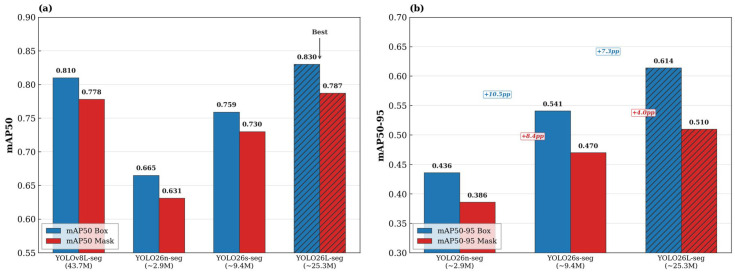
Multi-architecture comparison on the QHAWAY dataset. (**a**) Comparison of mAP50 Box and Mask for YOLOv8L-seg and the YOLO26 family; blue and red bars represent mAP50 Box and mAP50 Mask, respectively. (**b**) mAP50-95 scaling study across YOLO26 variants; blue annotations indicate percentage-point gains in Box and red annotations indicate gains in Mask between consecutive model scales. Red borders and diagonal hatching denote the best-performing model (YOLO26L-seg).

To assess the impact of architectural innovations on the QHAWAY dataset, a comparative evaluation was conducted incorporating the recently published YOLO26 model family [24,44]. YOLO26 introduces several advances pertinent to instance segmentation: NMS-free end-to-end inference, removal of Distribution Focal Loss (DFL), ProgLoss with Small-Target-Aware Label Assignment (STAL) for enhanced detection of underrepresented classes, the MuSGD optimiser, and a semantic segmentation loss that improves mask quality [44]. Three YOLO26-seg variants (nano, small, large) were trained on the identical QHAWAY hybrid dataset using the same three-phase progressive strategy and advanced augmentation (mixup 0.15, copy-paste 0.3, erasing 0.15, cosine learning rate schedule). Table 9 presents the comparative results, and Figure 9 provides a visual synthesis.

Table 10 presents the per-class comparison between YOLOv8L-seg and YOLO26L-seg.

YOLO26L-seg achieved the highest aggregate performance (mAP50 Box 0.829, mAP50 Mask 0.788), surpassing YOLOv8L-seg by +1.9 pp in Box and +1.0 pp in Mask. The recall improvement (+2.7 pp) indicates that YOLO26L-seg identifies a larger proportion of present objects, which is particularly consequential for safety-critical ADAS applications in which missed detections carry substantially higher risk than false positives. The YOLO26 scaling study reveals monotonically consistent improvement from nano to large variants, with the nano-to-large gap of +16.5 pp in mAP50 Box and +15.6 pp in mAP50 Mask confirming that model capacity remains a dominant factor for the 12-class QHAWAY task at 1024-pixel resolution.

### 4.7. Real-Time Performance

The QHAWAY ADAS system sustained processing rates between 19.2 and 25.4 FPS during operational validation on an NVIDIA RTX 5080 GPU (16 GB). Mean latency was 18–19 ms per frame, distributed across YOLOv8L-seg inference (≈15 ms), ByteTrack tracking (2 ms), distance estimation (1 ms), and alert management (1 ms). This configuration delivers latency within the human perception–reaction window stipulated for forward collision warning systems [14].

### 4.8. Representative Detections and Operational Validation

**Figure 10:**
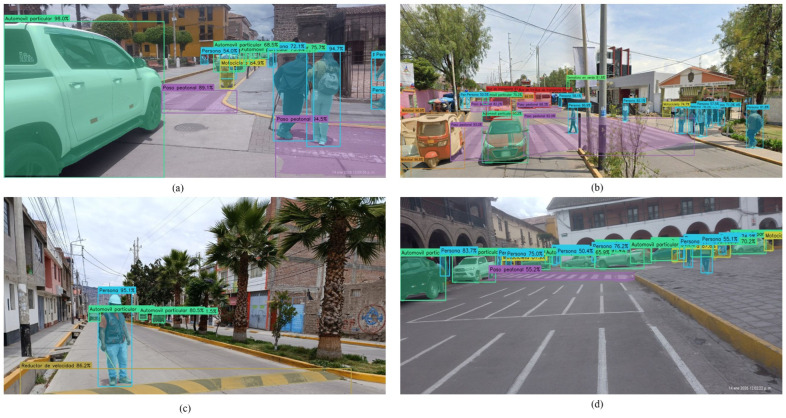
Representative QHAWAY ADAS detections in Ayacucho urban scenarios: (**a**) person with reduced mobility crossing a deteriorated crosswalk in the historic centre, demonstrating detection of vulnerable road users on narrow colonial streets; (**b**) mixed-traffic scene near UNSCH with simultaneous detection of mototaxis, buses, motorcycles, persons, crosswalks, and traffic lights; (**c**) speed bump with inadequate dimensions detected alongside a pedestrian and private vehicles; (**d**) high pedestrian and vehicular density in the historic centre, demonstrating robust detection under heavily overlapping instances.

**Figure 11:**
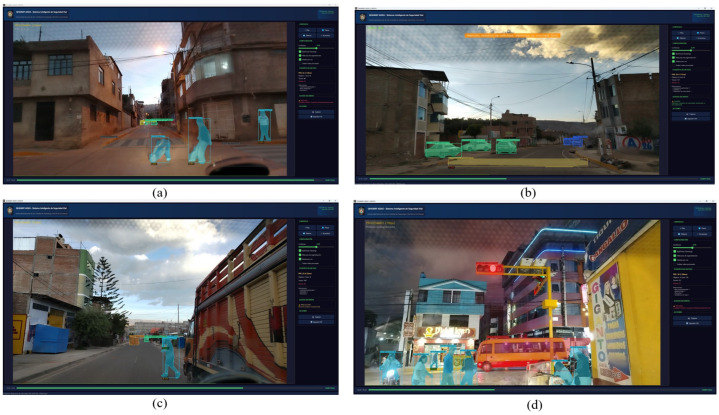
QHAWAY ADAS graphical interface during operational validation on Ayacucho streets: (**a**) DANGER-level alert triggered by two children who crossed unexpectedly; pinhole distance estimates of 5.6 m and 3.8 m (FPS: 22.1); (**b**) speed bump detection with anticipatory alert at 1.7 m on an unsigned road; (**c**) distracted pedestrian with mobile phone, detected upon emerging from behind a parked truck at 4.1 m; (**d**) simultaneous management of a red traffic light (confidence: 0.84) with five pedestrians in a nighttime scene (FPS: 19.2).

Operational validation was conducted under naturalistic driving conditions without any staged or provoked scenarios; all recorded events represent authentic traffic situations. During the validation campaign, several spontaneous events of high demonstrative value were captured, including the detection of two children crossing unexpectedly to retrieve a ball, a distracted pedestrian consulting a mobile device while emerging from behind a parked truck, and a red traffic light alert in a nighttime scene with multiple proximate pedestrians. The incidental occurrence of these events within a relatively brief validation window underscores the frequency and severity of routine road hazards in Ayacucho, reinforcing the practical necessity of a system such as QHAWAY. Figure 10 presents representative model detections, and Figure 11 illustrates the graphical user interface during real-time operation.

### 4.9. Distance Estimation: Geometric Validation

The operational accuracy of the pinhole distance estimator was assessed through a systematic geometric validation employing independent metric references inherent to the study environment. Reference measurements were derived from: (i) standard concrete pavement slab joints (3.0 m × 3.0 m grid), (ii) carriageway width (6.0 m, verified by the corresponding author acting in the capacity of certified civil engineer), and (iii) Toyota Hilux (Toyota Motor Corporation, Toyota, Japan) vehicle geometry (wheelbase 3085 mm; front bumper to camera distance ≈ 2.0 m).

Figure 11a documents two pedestrians at varying longitudinal and lateral positions relative to the vehicle trajectory. The child retrieving a ball was positioned approximately on the vehicle centreline at a longitudinal distance of 2.5 m in a crouching posture (apparent height ≈ 0.70 m). Applying the inverse pinhole equation:

(4) $$D_{\mathrm{real}}=D_{\mathrm{est}}\times \frac{H_{\mathrm{apparent}}}{H_{\mathrm{ref}}}=5.6\times \frac{0.70}{1.65}=2.37\;m$$

This corrected estimate of 2.37 m is consistent with the independent geometric ground-truth of 2.5 m (residual: 0.13 m), confirming that the model operates correctly and that the displayed overestimation is entirely attributable to the height-reference violation caused by the crouching posture. The adjacent standing pedestrian, laterally displaced approximately 1.0 m from the centreline at the same longitudinal distance, was estimated at 3.8 m. The lateral offset introduces a perspective angle of θ=arctan(1.0/2.5)≈21.8° and a true Euclidean camera distance of $\sqrt{2.5^{2}+1.0^{2}}=2.69$ m, explaining the residual overestimation of 1.11 m.

Figure 11b documents a non-conforming speed bump on an unmarked road. The device height (0.11 m, field-measured) marginally exceeds the reference value (0.10 m) and a road curvature of approximately 15° biases the apparent height slightly; QHAWAY estimated 1.7 m against a geometric reference of ≈2.0 m (residual: 0.3 m). Figure 11c presents a distracted pedestrian (height ≈ 1.60 m) emerging from behind a parked cargo truck. QHAWAY estimated 4.1 m; the corrected geometric reference yields:

(5) $$D_{\mathrm{real}}=4.1\times \frac{1.60}{1.65}=3.98\;m\approx 3.9\;m$$

with a residual of 0.2 m—the smallest error across all validated frames. Table 11 summarises the complete geometric validation results.

The distance estimation module serves a zone-classification function rather than a precision-ranging objective. All observed errors are geometrically predictable and operationally compensated by the independent TTC criterion; the system generates conservative (overestimating) failures in all cases, constituting a fail-safe behaviour. It should be noted that a formal quantitative validation with a calibrated laser rangefinder or LiDAR sensor was not performed in the present study, as such instrumentation is not commercially available in Ayacucho and sourcing it within the editorial revision period was not feasible. This systematic validation is designated as a primary priority for future work (Section 6).

### 4.10. Grad-CAM Explainability Results

**Figure 12:**
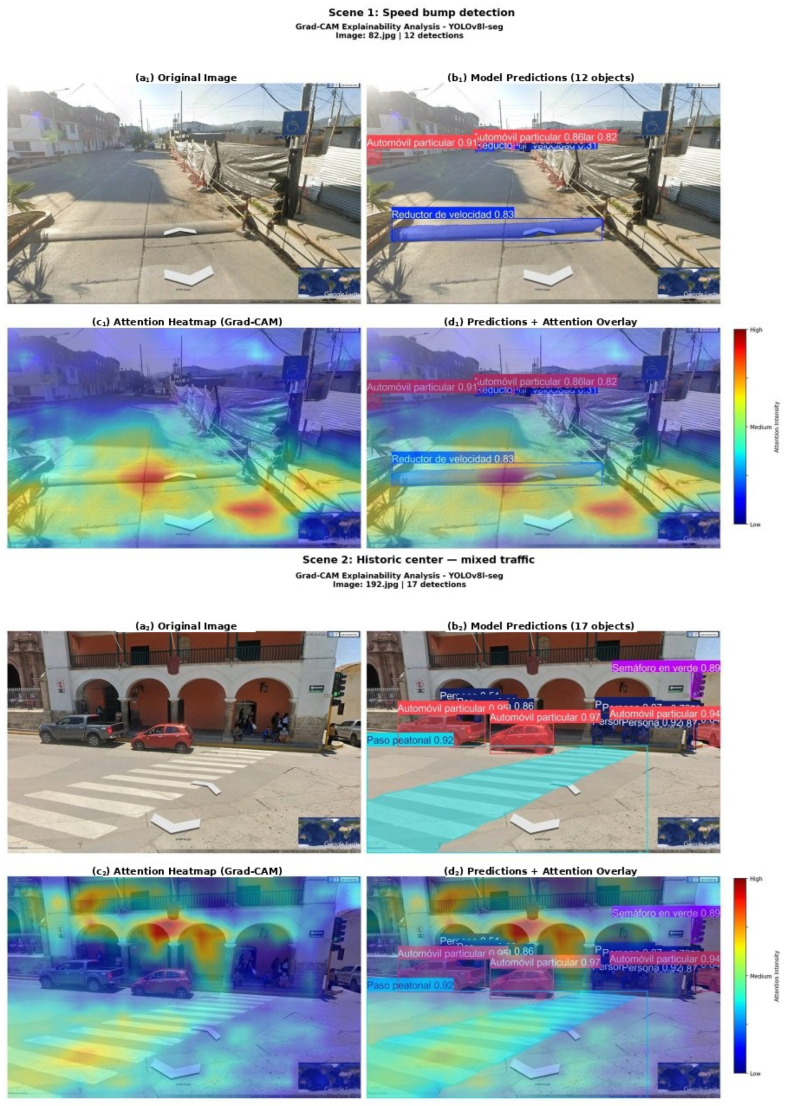
Grad-CAM explainability analysis of YOLOv8L-seg. Each scene presents four panels: original image, model predictions with confidence scores, attention heatmap (red = high activation, blue = low), and predictions overlaid on the attention map. Scene 1 (panels (**a_1_**–**d_1_**)): speed bump detection with attention concentrated on the device surface and surrounding vehicles. Scene 2 (panels (**a_2_**–**d_2_**)): mixed traffic in the historic centre with activation on vehicles, crosswalk, and traffic light, confirming that the model attends to safety-critical elements rather than to architecturally rich background structures. Class labels retain the original Spanish-language nomenclature from the annotated dataset (e.g., *Automóvil particular* = private car, *Persona* = person, *Reductor de velocidad* = speed bump).

**Figure 13:**
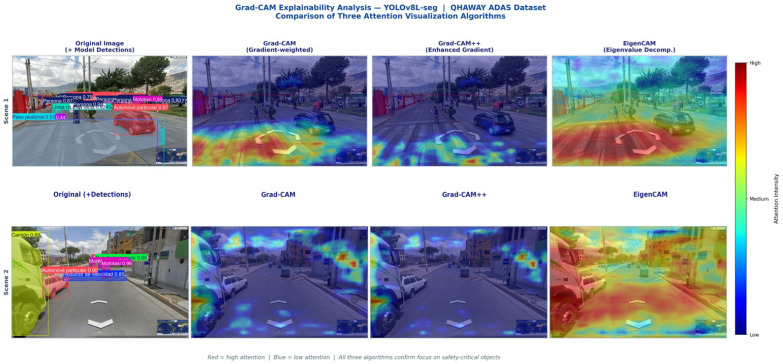
Comparative Grad-CAM explainability analysis employing three attention visualisation algorithms applied to two representative Ayacucho scenes. Columns from left to right: original image with model detections; Grad-CAM (gradient-weighted channel activation); Grad-CAM++ (enhanced gradient weighting for multi-instance scenes); EigenCAM (eigenvalue decomposition of feature maps, gradient-free). Scene 1 (mototaxi, crosswalk, and mixed traffic): all three algorithms converge on the carriageway and pedestrian crossing area as the primary attention locus, with activation concentrated on the mototaxi and vehicle contours. Scene 2 (informal urban corridor with bus, mototaxi, private car, speed bump, and truck): Grad-CAM and Grad-CAM++ concentrate activation on the vehicle cluster and the non-conforming speed bump surface in the foreground, whilst EigenCAM additionally highlights the road surface and lateral building facades. The consistent cross-algorithm agreement across both scenes provides strong evidence that the learned representations are semantically grounded in safety-critical objects rather than spurious background features of the Andean urban environment. Class labels in the detection column retain the original Spanish-language nomenclature from the annotated QHAWAY dataset (e.g., *Persona* = person, *Mototaxi* = three-wheeled motorised vehicle, *Reductor de velocidad* = speed bump, *Automóvil particular* = private car).

Figure 12 presents the original Grad-CAM explainability analysis for two representative scenes from the Ayacucho dataset. The attention heatmaps reveal that the model concentrates high-activation regions (red) on safety-critical objects, while background structures (buildings, sky, vegetation) receive consistently low activation (blue). Speed bumps generate activation in the lower third of the frame, geometrically consistent with their ground-level positioning. In the mixed-traffic scene, maximum activation zones coincide with vehicles and the crosswalk area, confirming that the model is not distracted by the rich architectural texture of the UNESCO-designated environment. Figure 13 extends the explainability analysis through a comparative evaluation of three complementary algorithms—Grad-CAM, Grad-CAM++, and EigenCAM—applied to two additional scenes representative of the Andean urban context.

## 5. Discussion

### 5.1. Informal Infrastructure: A Quantifiable Hazard

The field survey finding that 96% of speed bumps fail to comply with MTC Directive No. 01-2011-MTC/14 carries two significant implications. First, it validates the inclusion of speed bumps as a high-priority alert class: drivers unfamiliar with a given route lack reliable anticipatory cues for these devices. Second, it reveals a structural gap in the computer vision literature: all reviewed studies [20,26,27] target standardised devices, whereas the QHAWAY dataset is the first to incorporate and quantify non-conforming artisanal speed bumps as an independent detection class. The mAP50 Box of 0.923 for speed bumps—despite their substantial geometric variability—demonstrates that YOLOv8L-seg at 1024 px can successfully learn the visual signature of informal devices from 1847 local training instances. The marginally lower mask precision (0.897) reflects the irregular contour variability between semicircular and trapezoidal profiles.

### 5.2. Mototaxi: First Instance Segmentation Benchmark

The mototaxi achieved mAP50 Box of 0.769 and mAP50 Mask of 0.738, which to the best of the authors’ knowledge constitutes the first published instance segmentation evaluation of this vehicle class in an indexed journal. The present results extend the bounding-box detection benchmark reported in the group’s prior work [8] to mask-level evaluation, enabling more precise spatial analysis of this locally dominant vehicle category. The research group’s complementary ADAS study, Urbina-Dominguez et al. [45], addressed vertical traffic sign recognition (14 classes, bounding box) with voice alerts for the same Andean heritage city context, whilst the present work extends the scope to instance segmentation of horizontal infrastructure and vulnerable road users with monocular distance estimation.

### 5.3. Operational Reliability of Distance Estimation

The pinhole distance estimator implemented in QHAWAY fulfils a zone-classification function rather than a precision-ranging objective. The geometric validation conducted in the present study confirms that all observed estimation errors are attributable to identifiable physical causes—height-reference violations arising from non-standard postures, lateral displacement from the vehicle centreline, and marginal stature deviation from the reference value—rather than to algorithmic failures. Crucially, all observed deviations are conservative (overestimating), constituting a fail-safe failure mode for a safety-critical system: the system never underestimates the distance to a hazard. The sensor-agnostic character of the pinhole approach—requiring only a standard camera and known per-class heights—renders it particularly appropriate for ADAS deployment in developing-country contexts where specialised ranging sensors (stereo cameras, LiDAR, radar) are unavailable or economically impractical. Published validation studies confirm that the pinhole model achieves mean absolute errors below 2.5 m for urban driving distances up to 30 m [12], compatible with the operational zone boundaries (3 m danger, 7 m caution) defined in the present system. The primary contributions of this work reside in dataset construction, instance segmentation evaluation, and informal infrastructure characterisation; the distance estimation module constitutes a functional integration component rather than a metrological contribution.

### 5.4. Architectural Insights from the YOLO26 Comparison

The per-class comparison (Table 10) establishes that YOLO26L-seg improves upon YOLOv8L-seg in 9 of 12 classes for box detection and 7 of 12 for mask segmentation. The most pronounced gains occur in truck (+9.4 pp Box, +19.1 pp Mask), straight line (+8.7 pp Box, +6.9 pp Mask), and mototaxi (+5.2 pp Box). These improvements are attributable to the ProgLoss and STAL mechanisms of YOLO26, which systematically enhance detection of underrepresented and small-scale classes [44]. The truck class presents particularly pronounced intra-class visual variance: with only 177 training instances, the local fleet encompasses cargo trucks, tankers, tipper trucks, and canvas-covered vehicles, each exhibiting markedly different silhouettes and surface textures. This heterogeneity compounds the inherent difficulty of the limited training set, making the +19.1 pp mask gain attributable to YOLO26’s ProgLoss particularly salient. The person class—the highest-priority target for ADAS safety—improved by +2.3 pp Box and +2.0 pp Mask, a meaningful gain for a class with 4608 validation instances where marginal improvements are structurally harder to achieve. The mototaxi box detection gain of +5.2 pp (0.769 → 0.821) is especially significant for the local operational context, though the marginal mask performance decrease (−2.8 pp) suggests that YOLO26’s segmentation head handles the irregular mototaxi contour differently from YOLOv8. Conversely, speed bump box detection decreased by −4.0 pp (0.923 → 0.883), and straight-right line experienced the largest decline (−12.5 pp). These losses may reflect the trade-off inherent in YOLO26’s NMS-free end-to-end architecture, which eliminates the post-processing flexibility that benefited high-confidence, geometrically regular classes in YOLOv8. In aggregate, YOLO26L-seg achieves superior overall performance (mAP50 Box 0.829 vs. 0.810, mAP50 Mask 0.788 vs. 0.778) with markedly fewer parameters (≈25.3 M vs. 43.7 M). The recall improvement (+2.7 pp) is especially germane for safety-critical applications where missed detections carry higher risk than false positives. The YOLO26 scaling study (nano: 0.665, small: 0.759, large: 0.829 mAP50 Box) demonstrates consistent monotonic gains with increasing model capacity, furnishing practical deployment guidance across heterogeneous hardware configurations.

## 6. Limitations

*Flat road assumption in the pinhole model.* The pinhole camera model presupposes a flat road surface, a constant mounting height of 1.50 m, and known per-class object heights. The steep gradients present in the study road network (up to 12%), vehicle pitch during braking, and within-class height variation introduce estimation error. Published studies report mean absolute errors below 2.5 m for this model under urban conditions [12], adequate for operational zone classification (3 m/7 m boundaries) but insufficient for centimetre-precision applications.

*Absence of formal instrumented distance validation.* The pinhole distance estimator was not subjected to a formal quantitative validation against a calibrated reference instrument. Operational assessment relied on geometric consistency between displayed distances and independent metric references (pavement slab grid, carriageway width, vehicle geometry). Whilst the observed distances were consistent with ranges reported in the literature for monocular pinhole estimation [12,15], a systematic evaluation with a portable laser rangefinder or LiDAR sensor across controlled distances, object classes, and lighting conditions remains pending. This limitation reflects a logistical constraint specific to the study territory: Ayacucho is a geographically isolated high-altitude city (2761 m a.s.l.) where calibrated metrology instruments are not commercially available locally and cannot be sourced within the timeframe imposed by the editorial revision process. This systematic validation is designated as the primary priority for future work.

*Geometric variability of artisanal speed bumps.* The catalogued informal speed bumps exhibit highly heterogeneous geometries (heights between 0.08 and 0.21 m, semicircular profiles with varying curvatures, cobblestone versus asphalt surfaces), amplifying intra-class variance. This variability simultaneously affects detection confidence and distance estimation accuracy, as the reference height ($H_{\mathrm{real}}=0.10$ m) represents a single nominal value for a geometrically diverse class.

*Mototaxi class representation.* With 357 local training instances, the mototaxi class attains adequate but sub-optimal performance (mAP50 Mask 0.738). The training set covers the predominant mototaxi configuration in Ayacucho but does not represent regional variants with alternative roofing materials (canvas, acrylic, metal) or divergent body configurations.

*Validation on recorded sequences without driver physiological response assessment.* The validation protocol does not evaluate actual driver responses to voice alerts: braking reaction time, voice message intelligibility under real road noise conditions, or additional cognitive load generated by alerts.

*Atmospheric conditions at 2761 m a.s.l.* Operation at altitude engenders specific optical conditions: elevated UV radiation accelerating the degradation of road marking pigments, abrupt thermal variations, and highland fog. Although the dataset encompasses captures under varied meteorological conditions, no systematic evaluation was conducted under hail or dust conditions.

## 7. Conclusions

This paper has presented QHAWAY, an instance-segmentation-based advanced driver assistance system for vulnerable road users in the informal Andean urban environment of Ayacucho, Peru. Seven principal conclusions are advanced. First, the hybrid dataset of 25,602 images with 127,525 instances across 12 classes, assembled through four complementary acquisition methods across the five urban districts of the Huamanga province, is the first dataset representing the informal Andean road environment, incorporating the mototaxi as an independent class. Second, the three-phase progressive training strategy with increasing resolution (640, 800, 1024 px) yielded a cumulative gain of +24.9 pp in mAP50 Mask over the Phase 1 baseline, with the elevation to 1024 px contributing the largest mask improvement (+14.5 pp). Third, YOLOv8L-seg attained mAP50 Box of 0.810 and mAP50 Mask of 0.778; the multi-architecture comparison identified YOLO26L-seg as the best-performing model with mAP50 Box of 0.829 and mAP50 Mask of 0.788, operating in real time at 19.2–25.4 FPS on an NVIDIA RTX 5080 GPU. Fourth, the YOLO26 scaling study (nano, small, large) demonstrated consistent monotonic performance gains with increasing model capacity, providing practical guidance for deployment across heterogeneous hardware configurations. Fifth, the first instance segmentation benchmark for the Peruvian mototaxi (mAP50 Box 0.769, mAP50 Mask 0.738 with YOLOv8L-seg) establishes a quantitative baseline for future localised ADAS research. Sixth, the systematic field survey documented that 96% of audited speed bumps fail to comply with MTC Directive No. 01-2011-MTC/14; the model achieved mAP50 Box of 0.923 for this class—the highest across the entire 12-class taxonomy. Seventh, a comprehensive Grad-CAM analysis encompassing three complementary algorithms (Grad-CAM, Grad-CAM++, EigenCAM) confirmed the semantic coherence of learned representations, with safety-critical attention patterns on proximate road actors and ground-level activation for speed bump detection.

### Future Work

*Formal quantitative validation of pinhole distance estimation.* A systematic validation campaign employing a portable calibrated laser rangefinder or LiDAR sensor—instrumentation currently unavailable in the study territory but targeted for acquisition in the subsequent phase of this research—will be conducted across multiple object classes, distance ranges, lighting conditions, and road gradients. This campaign will establish the empirical mean absolute error of the QHAWAY distance module and enable quantitative comparison with published monocular and stereo ADAS benchmarks, including those reported by Dai et al. [12] and Chaman et al. [13].

*Road gradient compensation via IMU.* Incorporating real-time inertial measurement unit data to estimate instantaneous vehicle inclination will enable dynamic correction of the tilt angle θ in Equation 2, reducing pinhole estimation error on the steep gradient segments documented in the study area.

*Stereo camera calibration.* Adding a calibrated stereo camera to the instrumentation setup will provide continuous depth reference during driving, enabling simultaneous dynamic validation of the pinhole estimator and automatic focal length adjustment under temperature or mechanical vibration changes.

*Synthetic data generation for mototaxi variants.* Generating photorealistic 3D models of mototaxi variants present in Peruvian cities will expand the class 11 training set without requiring additional field collection campaigns.

*Extension of the informal infrastructure survey.* The metric survey protocol will be extended to additional roads within Ayacucho and replicated in municipalities with documented mototaxi presence (Juliaca, Iquitos, Pucallpa), enabling assessment of model transferability across informal road contexts with different geometric and illumination characteristics.

*Biometric-instrumented driver response evaluation.* In situ validation during genuine driving will require instrumented protocols with participant drivers, recording braking reaction time, voice message comprehension under real road noise conditions, and associated cognitive load.

## Figures and Tables

**Table 1:** Reference real heights ($H_{\mathrm{real}}$) per class employed in the pinhole distance estimator.

ID	Class	$H_{\mathrm{real}}$ (m)	Source
0	Speed bump (device height)	0.10	MTC field survey
1	Crosswalk (stripe width)	0.30	MTC standard
4	Person	1.65	Peruvian adult anthropometry [31]
5	Motorcycle	1.10	Field measurement
6	Private car	1.50	Field measurement
7	Truck	3.20	MTC regulation
8	Bus	3.00	MTC regulation
10	Traffic light (lens diameter)	0.30	MTC standard
11	Mototaxi (with roof)	1.60	Field measurement

**Table 2:** Field survey results for informal speed bumps, benchmarked against MTC Directive No. 01-2011-MTC/14.

Criterion	MTC Standard	Observed (Mean)	Compliance (%)
Height	≤0.08 m	0.11 m	4
Cross-section	Trapezoidal	Semicircular	0
Vertical sign (≤100 m)	Mandatory	Absent	4
Surface marking	Mandatory	Absent or deteriorated	8
**Overall compliance**	–	–	**4**

**Table 3:** Class distribution in the original local Ayacucho dataset (4598 images, 10,701 instances).

ID	Class Name	Instances	%
0	Speed bump	1847	17.3
1	Crosswalk	412	3.9
2	Straight line	1203	11.2
3	Straight-right line	634	5.9
4	Person	2918	27.3
5	Motorcycle	891	8.3
6	Private car	1074	10.0
7	Truck	213	2.0
8	Bus	287	2.7
9	Green traffic light	344	3.2
10	Red traffic light	521	4.9
11	Mototaxi	357	3.3
	**Total**	**10,701**	**100.0**

**Table 4:** Composition of the QHAWAY hybrid dataset.

Source	Images	Instances	Classes
Original Ayacucho (local)	4598	10,701	12
BDD100K (subset)	14,211	78,932	8
BSTLD	5093	30,517	2
RLMD	1700	7375	2
**Hybrid total**	**25,602**	**127,525**	**12**

**Table 5:** Three-phase progressive training configuration with increasing input resolution.

Phase	Epochs	Resolution (px)	Batch Size	Learning Rate	Initial Weights
Phase 1	100	640 × 640	16	0.01	COCO pre-trained
Phase 2	150	800 × 800	8	0.001	Best from Phase 1
Phase 3	223	1024 × 1024	4	0.0005	Best from Phase 2

**Table 6:** Ablation study: incremental contribution of each training phase to model performance on the validation set.

Phase	Resol. (px)	mAP50 Box	mAP50 Mask	ΔBox	ΔMask
Phase 1 (640 px)	640	0.682	0.529	–	–
Phase 2 (800 px)	800	0.741	0.633	+5.9 pp	+10.4 pp
Phase 3 (1024 px)	1024	**0.810**	**0.778**	+6.9 pp	+14.5 pp
**Total gain, Phase 1 → Phase 3**				**+12.8 pp**	**+24.9 pp**

**Table 7:** Overall performance of YOLOv8L-seg (Phase 3, 1024 px, epoch 173) on the validation set.

Metric	mAP50	Precision	Recall	F1
Detection (Box)	0.810	0.885	0.724	0.796
Segmentation (Mask)	0.778	0.876	0.690	0.772

**Table 8:** Per-class performance of YOLOv8L-seg (mAP50) on the validation set (epoch 173).

Class	Val. Instances	mAP50 Box	mAP50 Mask
Speed bump	379	0.923	0.897
Straight-right line	72	0.898	0.898
Red traffic light	255	0.892	0.785
Person	4608	0.866	0.826
Green traffic light	251	0.861	0.828
Private car	2256	0.828	0.791
Motorcycle	1025	0.791	0.786
Mototaxi	566	0.769	0.738
Crosswalk	563	0.765	0.771
Bus	440	0.762	0.728
Truck	177	0.756	0.659
Straight line	109	0.610	0.628
**All classes**	**10,701**	**0.810**	**0.778**

**Table 9:** Multi-architecture comparison on the QHAWAY validation set. All YOLO26 models report the best checkpoint across 300 epochs at 1024 px; YOLOv8L-seg reports the best checkpoint at epoch 173.

Model	Best Ep.	mAP50 Box	mAP50 Mask	mAP50-95 Box	mAP50-95 Mask	P(B)	R(B)
YOLOv8L-seg	173	0.810	0.778	–	–	0.885	0.724
YOLO26n-seg	222	0.665	0.631	0.436	0.386	0.737	0.602
YOLO26s-seg	242	0.759	0.730	0.541	0.470	0.831	0.676
YOLO26L-seg	179	**0.829**	**0.788**	**0.614**	**0.510**	0.868	**0.751**

**Table 10:** Per-class performance comparison between YOLOv8L-seg (epoch 173) and YOLO26L-seg (epoch 179). Δ indicates the YOLO26L-seg gain (positive) or loss (negative) relative to YOLOv8L-seg. Bold values indicate the higher mAP50 per class.

Class	mAP50 Box			mAP50 Mask		
	v8L	26L	Δ	v8L	26L	Δ
Red traffic light	0.892	**0.935**	+4.3	0.785	**0.795**	+1.0
Person	0.866	**0.889**	+2.3	0.826	**0.846**	+2.0
Green traffic light	0.861	**0.899**	+3.8	**0.828**	0.824	−0.4
Speed bump	**0.923**	0.883	−4.0	**0.897**	0.882	−1.5
Private car	0.828	**0.853**	+2.5	0.791	**0.793**	+0.2
Truck	0.756	**0.850**	+9.4	0.659	**0.850**	+19.1
Mototaxi	0.769	**0.821**	+5.2	**0.738**	0.710	−2.8
Motorcycle	0.791	**0.820**	+2.9	0.786	**0.796**	+1.0
Straight line	0.610	**0.697**	+8.7	0.628	**0.697**	+6.9
Straight-right line	**0.898**	0.773	−12.5	**0.898**	0.773	−12.5
Crosswalk	0.765	**0.769**	+0.4	0.771	**0.776**	+0.5
Bus	0.762	**0.763**	+0.1	**0.728**	0.709	−1.9
**All classes**	0.810	**0.829**	+1.9	0.778	**0.788**	+1.0

**Table 11:** Geometric validation of the pinhole distance estimator across representative operational frames. $D_{\mathrm{geo}}$ denotes the independent geometric reference distance; $D_{\mathrm{est}}$ denotes the QHAWAY estimate; ε denotes the absolute residual.

Frame/Object	$D_{\mathrm{geo}}$ (m)	$D_{\mathrm{est}}$ (m)	ε (m)	Primary Cause of Deviation
(a) Child crouching	2.50	5.60	3.10	Height-reference violation ($H_{\mathrm{app}}=0.70$ m)
(a) Standing pedestrian	2.69 ^†^	3.80	1.11	Lateral displacement (θ≈21.8°)
(b) Speed bump	≈2.00	1.70	0.30	$H_{\mathrm{real}}>H_{\mathrm{ref}}$; road curvature
(c) Standing pedestrian	3.90	4.10	0.20	Minor height deviation (H=1.60 m vs. 1.65 m)

^†^ Euclidean camera distance, accounting for 1.0 m lateral offset.

## Data Availability

The original local Ayacucho dataset (4598 images, 10,701 instances) is available upon reasonable request to the corresponding author. The international dataset subsets (BDD100K, BSTLD, RLMD) are publicly accessible under their respective licences.

## Abbreviations

ADAS	Advanced Driver Assistance System
FCW	Forward Collision Warning
FPS	Frames per Second
Grad-CAM	Gradient-weighted Class Activation Mapping
IMU	Inertial Measurement Unit
MAE	Mean Absolute Error
mAP	Mean Average Precision
MTC	Ministry of Transport and Communications
NHTSA	National Highway Traffic Safety Administration
STAL	Small-Target-Aware Label Assignment
TTC	Time-to-Collision
TTS	Text-to-Speech
YOLO	You Only Look Once
